# Frailty Indices and Preoperative Clinical Factors for Predicting Myocardial Injury After Non-Cardiac Surgery (MINS) in High-Risk Geriatric Orthopedic Patients: A Machine-Learning Study

**DOI:** 10.3390/jcm15218285

**Published:** 2026-10-24

**Authors:** Dilek Kalaycı, Edanur Genç Turan, İmran Ceren, Fadime Bozduman Habip, Özlem Şen, Tuğba Aşkın, Çoşkun Ulucaköy

**Affiliations:** 1 Department of Anesthesiology and Reanimation, Dr. Abdurrahman Yurtarslan Ankara Oncology Training and Research Hospital, University of Health Sciences, 06200 Ankara, Turkey; edanrgenc@gmail.com (E.G.T.); dr.tugba.askin@gmail.com (T.A.); 2 Department of Cardiology, Dr. Abdurrahman Yurtarslan Ankara Oncology Training and Research Hospital, University of Health Sciences, 06200 Ankara, Turkey; drimranceren@gmail.com (İ.C.); drfadimebozduman@gmail.com (F.B.H.); 3 Department of Anesthesiology and Reanimation, Republic of Turkey Ministry of Health Muğla Training and Research Hospital, 48000 Muğla, Turkey; turanozlem1@yahoo.com.tr; 4 Department of Orthopedics and Traumatology, Dr. Abdurrahman Yurtarslan Ankara Oncology Training and Research Hospital, University of Health Sciences, 06200 Ankara, Turkey; coskunulucakoy@gmail.com

**Keywords:** frailty, modified frailty index, geriatric orthopedic surgery, machine learning, perioperative risk, myocardial injury

## Abstract

**Background/Objectives**: Myocardial injury after non-cardiac surgery (MINS) is a common, often asymptomatic complication in geriatric orthopedic patients that increases postoperative mortality. The Revised Cardiac Risk Index (RCRI) may not adequately capture the age-related loss of the physiological reserve. We investigated whether frailty indices (mFI-5, mFI-11) improve machine-learning prediction of preoperative MINS risk, and compared an exploratory perioperative model with ASA and RCRI. **Methods**: In this single-center retrospective study, 656 patients aged ≥ 65 years who underwent major orthopedic surgery with postoperative ICU monitoring and troponin surveillance were analyzed; MINS occurred in 82 (12.5%). The models were as follows: Model 1 (preoperative baseline), Model 2 (+mFI-5), Model 3 (+mFI-11), and an exploratory perioperative model, Model 4 (+intraoperative vasopressor use and postoperative mechanical ventilation). AUCs on a 30% hold-out set were compared with ASA/RCRI using the paired DeLong test, with Bonferroni adjustment across 15 comparisons. A sensitivity analysis with an identical classifier and predictor set isolated the contribution of the frailty indices. **Results**: Frailty indices did not provide significant incremental value: adding mFI-5 or mFI-11 changed the AUC only marginally (0.5744 to 0.5828 and 0.5881; all *p* > 0.5). Preoperative models (hold-out AUC 0.6005–0.6935) did not differ significantly from ASA or RCRI after Bonferroni correction. Model 4, incorporating intra- and postoperative variables, reached an AUC of 0.7584, significantly higher than ASA (adjusted *p* = 0.002) but not RCRI (adjusted *p* = 0.268). **Conclusions**: With the algorithm and predictor set held constant, no incremental value of frailty indices was demonstrable, and no model significantly outperformed RCRI after multiplicity correction. The numerically higher AUC of Model 3 versus Model 2 cannot be attributed to frailty indices, as the models differed in both predictors and algorithm. Power was limited; these findings are hypothesis-generating. Whether frailty indices improve prediction needs to be tested in external cohorts large enough to detect it.

## 1. Introduction

Myocardial injury after non-cardiac surgery (MINS) is a serious complication characterized by an elevation in postoperative cardiac troponin (cTn) levels following a surgical intervention, occurring independently of ischemic symptoms or ECG findings [1]. The majority of MINS cases are silent [2]. In the literature, some meta-analyses report a prevalence above 20%, and it is considered one of the major causes of postoperative mortality [3]. It has been reported that 30-day mortality increases approximately 11-fold in patients who develop MINS following major orthopedic surgery [4].

The Revised Cardiac Risk Index (RCRI) remains the most widely used tool in perioperative cardiac risk assessment. However, RCRI focuses solely on organ failures and fails to reflect the loss of reserve associated with aging or the acute inflammatory response induced by orthopedic surgery. For these reasons, the performance of RCRI in predicting MINS is limited [5,6], and more sensitive tools are needed for individualized risk management.

Frailty is a multidimensional geriatric syndrome that develops as a result of the loss of physiological reserve across systems with aging, and it is an important risk factor reflecting biological reserve beyond chronological age [7,8]. The prevalence of frailty exceeds 50% in patients over 85 years of age [7] and intraoperative and postoperative complications are observed more frequently in these patients [9]. It affects 23% of patients undergoing elective orthopedic surgery and 53% of patients undergoing emergency hip fracture surgery [8,9]. Geriatric hip fracture patients constitute the highest-risk group for MINS due to advanced age, high comorbidity burden, decreased physiological reserve, and the inflammatory response induced by surgery and trauma [10]. Nevertheless, the additional prognostic contribution of frailty scores to the prediction of MINS in the geriatric orthopedic population remains unclear. In the literature, the relationship between frailty and cardiac risk has mostly been examined using single frailty indices. Joseph et al. demonstrated the contribution of frailty to surgical risk prediction but did not focus on MINS [11]; the VISION study, on the other hand, established the prognostic role of MINS but did not evaluate frailty scales [12]. To our knowledge, no machine learnings of the MINS study has yet compared mFI-5 and mFI-11 within the same cohort of a geriatric orthopedic population. This study aims to compare the discriminative power of mFI-5, which offers ease of use in clinical practice, against mFI-11, which reflects comorbidity burden in greater detail.

Moreover, traditional statistical methods in perioperative risk modeling may not fully capture the complex interactions among age, comorbidity burden, biomarkers, and frailty indices [13]. Machine learning can capture complex, non-linear relationships among clinical variables and may complement traditional statistical models.

The primary aim of this study was to compare the discriminative performance of baseline preoperative clinical and biochemical variables (Model 1) against sequentially developed machine learning models that integrate modified frailty indices—mFI-5 (Model 2) and mFI-11 (Model 3)—for predicting MINS in elderly patients undergoing major orthopedic surgery. Secondarily, we aimed to compare an exploratory model (Model 4), obtained by incorporating additional parameters reflecting the perioperative dynamic course, with conventional risk scores based on the American Society of Anesthesiologists (ASA) and RCRI.

## 2. Materials and Methods

### 2.1. Study Design and Ethical Approval

This study is a single-center, retrospective observational cohort study including data from patients aged 65 years and older who underwent major orthopedic surgery between January 2021 and December 2023 and were subsequently followed in the intensive care unit for close hemodynamic monitoring and routine clinical care due to high-risk profiles in the postoperative period. The study protocol was approved by *the Clinical Research Ethics Committee of Dr. Abdurrahman Yurtarslan Ankara Oncology Training and Research Hospital* (Ethics Committee No: 2026-04/85) and registered on ClinicalTrials.gov (NCT07566013); the study was conducted in accordance with the principles of the Declaration of Helsinki.

### 2.2. Patient Population and Primary Outcome

Patients aged 65 years and older who underwent hip fracture repair, total knee or hip arthroplasty, revision surgery, or upper extremity surgery; had complete preoperative clinical and laboratory data (hemoglobin, creatinine, and albumin); and had at least one high-sensitivity cardiac troponin T (hs-cTnT) measurement within the first 72 postoperative hours were included in the study. Troponin measurements were performed as part of routine clinical care. Patients with a history of preoperative acute myocardial infarction or a preoperative baseline hs-cTnT ≥ 65 ng/L, those with end-stage renal disease requiring dialysis, and those with missing critical preoperative data or incomplete postoperative troponin follow-up were excluded; a total of 656 patients were included in the analysis. Patients with a baseline hs-cTnT ≥ 65 ng/L were excluded because the VISION criterion of any postoperative value ≥ 65 ng/L cannot distinguish acute perioperative injury from pre-existing chronic myocardial injury in this subgroup. Patients with a baseline hs-cTnT below 65 ng/L were retained, and MINS was ascertained from the postoperative change relative to baseline. Preoperative and postoperative troponin measurements were performed using the (Roche Diagnostics, Mannheim, Germany) Elecsys hs-cTnT assay (99th percentile upper reference limit: 14 ng/L). The primary outcome was MINS developing within the first 72 h after surgery. MINS was defined, regardless of the presence of ischemic symptoms, according to the VISION study criteria [12], as a postoperative hs-cTnT concentration of 20 to <65 ng/L with an absolute change of ≥5 ng/L from the preoperative baseline, or any postoperative hs-cTnT concentration ≥ 65 ng/L, provided that non-cardiac causes capable of elevating troponin (severe sepsis, massive pulmonary embolism) were excluded.

### 2.3. Data Source, Variables, and Data Preprocessing

Data were obtained through retrospective review of the institution’s hospital information management system and anesthesia follow-up forms. The preoperative variables recorded were age, sex, ASA physical status score, surgical urgency, type of surgical procedure, chronic comorbidities (hypertension, diabetes, atrial fibrillation, COPD, dementia/Alzheimer’s disease, heart failure, coronary artery disease), and basic laboratory parameters (hemoglobin, serum creatinine, serum albumin). Ischemic heart disease was defined as a history of myocardial infarction and/or coronary artery disease/prior cardiac surgery recorded in hospital files. Preoperative medication use (Supplementary Table S1) was extracted from medication records, and prior CABG and PCI/stent from free-text comorbidity notes. Stent type, time since PCI, and perioperative interruption of antiplatelet therapy were not systematically recorded. Prior CABG and PCI/stent are reported descriptively and were not used as model inputs.

Frailty was quantified with the Modified Frailty Indices (mFI-5 and mFI-11) [14,15] to express patients’ physiological reserve. The Revised Cardiac Risk Index (RCRI) [16] and the American Society of Anesthesiologists Physical Status classification (ASA-PS) [17] were used only as comparators and were not entered into any machine learning model. All scales were calculated retrospectively from preoperative data obtained from the hospital information management system and anesthesia monitoring forms. The mFI-5 (range 0–5) assigns one point each for diabetes mellitus, hypertension, heart failure, COPD/pneumonia, and functional dependence. The mFI-11 (range 0–11) comprises these five items plus previous myocardial infarction, history of coronary artery disease/cardiac surgery, peripheral vascular disease, cognitive impairment, cerebrovascular disease, and previous stroke. Each component was scored 1 if present and 0 if absent, and the scores were summed; non-normalized raw total scores were used in all analyses. The RCRI (range 0–6) assigns one point each for high-risk surgery, ischemic heart disease (defined as above), heart failure, cerebrovascular disease, insulin-treated diabetes, and serum creatinine > 2 mg/dL. The ASA-PS (classes I–V) was taken from the anesthesia record: I, normal healthy patient; II, mild systemic disease; III, severe systemic disease; IV, severe systemic disease that is a constant threat to life; V, moribund patient not expected to survive without the operation.

All preprocessing steps were integrated into the cross-validation framework to prevent data leakage. Missing values in continuous variables were imputed using K-Nearest Neighbor (KNN, k = 5) imputation, and categorical variables were imputed using mode imputation. Categorical features were transformed using drop-first One-Hot Encoding to reduce multicollinearity. All transformation parameters were learned solely from the training set and then applied to the validation/test subsets.

### 2.4. Variable Selection Strategy and Statistical Analysis

Variable selection during model development was performed in three stages. Initially, from a pool of 42 candidate variables, model-configuration-specific exclusion criteria were applied in the first stage. Accordingly, the ASA and RCRI scores, being comparator measures, were not included in any model. The frailty index not belonging to the respective model (e.g., mFI-11 in the mFI-5 model; mFI-5 in the mFI-11 model), the sub-clinical components of the frailty score added to the model (to prevent redundant information entry), and variables unknown preoperatively (vasopressor (VP) and mechanical ventilation (MV) support requirements; reserved solely for Model 4) were removed from the candidate pool. In the second stage, the remaining variables were subjected to Variance Inflation Factor (VIF) analysis for multicollinearity risk, and variables with VIF > 5.0 were eliminated. This threshold was preferred as it represents a stricter approach compared to the VIF = 10 criterion [18,19]. Because they constitute the study’s core research question, the mFI-5 and mFI-11 indices were excluded from this VIF elimination process. In the third stage, variables that did not provide a significant contribution to the model, based on the intrinsic importance scores and permutation mean decrease scores calculated in the inner loop within the training dataset, were iteratively eliminated, and the final variable set for each model was established.

The conformity of continuous variables to a normal distribution was assessed using the Shapiro–Wilk test and visual methods (histogram, Q-Q plot). Normally distributed data were summarized as mean ± standard deviation, and non-normally distributed data as median (interquartile range, IQR). Categorical variables were expressed as frequency and percentage. Comparisons between groups were performed, as appropriate, using Student’s *t*-test, the Mann–Whitney U test, Pearson’s chi-square test, or Fisher’s exact test. The threshold for statistical significance was set at *p* < 0.05.

### 2.5. Model Architecture and Development Protocol

To predict the risk of developing MINS and to progressively evaluate the contribution of clinical variables and frailty scales to the model, four different machine learning models were constructed. The cohort was randomly split 70/30 into training and hold-out test sets.

Model 1 (Baseline Preoperative Model): Excludes frailty indices and perioperative interventions, including only routine preoperative demographic, comorbidity, and laboratory parameters. Following the elimination processes, a total of 9 variables remained in the model.

Model 2 (mFI-5-Layered Preoperative Model): Constructed by adding the Modified Frailty Index-5 (mFI-5) score to the preoperative variables in Model 1. Variables constituting components of mFI-5 were removed to prevent multicollinearity. After VIF- and contribution-based elimination, a total of 10 variables remained in the model.

Model 3 (mFI-11-Layered Preoperative Model): Constructed by adding the Modified Frailty Index-11 (mFI-11) score to the preoperative variables in Model 1. The components of mFI-11 were removed from the model. Following these elimination processes, a total of 6 variables remained in the model.

Model 4 (Extended Perioperative Course Model): The most comprehensive model, encompassing, in addition to preoperative variables, the patient’s intraoperative/postoperative clinical course and the vasopressor (VP) and mechanical ventilation (MV) support requirements reflecting hemodynamic stress. Following these processes, a total of 12 variables remained in the model.

All four models were developed independently following the same nested cross-validation, algorithm selection, and calibration protocol, and were evaluated on the internal hold-out test set. Models 1–3 represent the primary aim of the study, namely preoperative risk prediction. Model 4, containing intraoperative/postoperative variables, falls outside the scope of preoperative decision support and is reported solely as an exploratory perioperative model intended to demonstrate the predictive contribution of VP use and MV requirement.

Because of the limited number of MINS events (*n* = 82), the following measures were used to mitigate overfitting and optimistic performance estimates. In the first stage, the LazyPredict library was used to screen a broad range of baseline classifiers on the training subset; the top three algorithms by ROC-AUC were combined with seven standard classifiers commonly used in clinical risk prediction (Logistic Regression, Random Forest, XGBoost, LightGBM, CatBoost, Support Vector Machines, Nearest Centroid), yielding a candidate pool of 10 models. The internal hold-out test set was not used at this stage. Following clinical pre-screening, multicollinearity was controlled using a fixed VIF threshold of ≤5.0, and feature selection was conducted via iterative permutation-importance pruning within the inner loop of nested cross-validation; wrapper methods such as recursive feature elimination were not used. In the second stage, 5-fold nested cross-validation was applied; each model was optimized in the inner loop with GridSearchCV over predefined grids using both standard and class-weighted configurations, and subjected to probability calibration. In the outer loop, the model with the highest mean ROC-AUC was selected as the final model and evaluated on the internal hold-out test set, which was not included in the model development process at any stage.

To isolate the incremental contribution of the frailty indices, a sensitivity analysis was performed in which the model architecture was kept fully comparable across models. All components of mFI-5 and mFI-11 were excluded from all three models. The three models used the same classifier (class-weighted Nearest Centroid) and the same candidate predictor pool; the only difference between them was the inclusion of no frailty index (comparable base model), the mFI-5 score (+mFI-5 model), or the mFI-11 score (+mFI-11 model). Nearest Centroid was chosen because it was the algorithm selected for Models 1 and 2, so that the frailty indices could be evaluated within the same framework as the primary baseline model; the CatBoost algorithm selected for Model 3 was not replicated in this analysis. To ensure an identical predictor set across the three models, VIF-based exclusion and importance-based iterative feature selection were intentionally omitted, resulting in models containing 25–26 variables each. These are diagnostic models designed to isolate the independent contribution of frailty, not candidate clinical models; the variable count should therefore not be evaluated against conventional events-per-variable (EPV) thresholds. All other preprocessing, nested cross-validation, and probability calibration procedures were identical to those used for the primary models. Performance was evaluated on the same internal hold-out test set, with pairwise differences in discrimination assessed using the paired DeLong test. Because this analysis was designed strictly to isolate the effect of frailty rather than to optimize predictive performance, no formal comparisons were made between the AUC values of these models and those of the primary models.

### 2.6. Performance Evaluation, Model Interpretability, and Software

The discriminative power of the models was evaluated using ROC-AUC, sensitivity, and specificity, and the optimal decision threshold was determined using the Youden Index. Calibration was assessed using the Brier score and calibration curves (reliability diagrams), with the 95% confidence intervals for the calibration intercept and slope, as well as for AUC were obtained by bootstrap resampling (1000 iterations) of the hold-out test set.; clinical benefit was assessed using Decision Curve Analysis. Model performances were compared with ASA and RCRI; differences between ROC curves were assessed using the DeLong test, and 95% confidence intervals were calculated using the bootstrap method (1000 iterations). Pairwise AUC comparisons among the four models were also performed using the DeLong test, and Bonferroni correction was applied across all 15 pairwise comparisons among the six candidates (Models 1–4, the ASA physical status classification, and RCRI); the full corrected results are presented in Supplementary Table S2. Model interpretability was evaluated using SHAP (SHapley Additive exPlanations) and permutation feature importance analysis. Native importance scores were derived using algorithm-specific metrics (PredictionValuesChange for CatBoost; Absolute Centroid Difference for Nearest Centroid) and are not directly comparable across model types; only permutation importance is comparable across algorithms. SHAP values were computed on the 30% hold-out test set for all models; these analyses were performed post hoc, strictly for model interpretation, and did not influence model selection, hyperparameter tuning, or feature elimination, all of which were finalized exclusively on the training subset prior to hold-out evaluation.

All data preprocessing, statistical analyses, and machine learning modeling were performed using Python (v3.12.3, Python Software Foundation, Wilmington, DE, USA; Spyder environment). Pandas (v3.0.3) and NumPy (v1.26.4) were used for data manipulation; statsmodels (v0.14.2, VIF) for multicollinearity control; and SciPy (v1.13.1, DeLong test) for the statistical significance of model comparisons. The machine learning pipeline (including imputation, scaling, nested cross-validation, hyperparameter optimization, and probability calibration) was built using scikit-learn (v1.8.0); XGBoost (v2.1.3), LightGBM (v4.6.0), and CatBoost (v1.2.10) libraries were used for algorithmic modeling and gradient boosting architectures. Model inter-pretability was provided using SHAP (v0.48.0),permutation feature importance was computed using scikitlearn’s permutation importance module and graphical representations using Matplotlib (v3.8.4) and Seaborn (v0.13.2).

## 3. Results

During the study period, a total of 800 patients were screened. Following evaluation, 144 patients were excluded: 53 with preoperative baseline hs-cTnT ≥ 65 ng/L, 19 with end-stage renal disease requiring dialysis, and 72 with missing critical preoperative data or incomplete postoperative troponin follow-up. Analyses were performed on a cohort of 656 patients (Figure 1).

The median age of patients who developed MINS was significantly higher than that of patients who did not [80.5 (IQR: 73.0–86.0) vs. 78.0 (IQR: 72.0–83.0) years, *p* = 0.048]. Congestive heart failure was significantly more prevalent in the MINS group (28.0% vs. 11.3%, *p* < 0.001), representing the strongest univariate association observed in this cohort; congestive heart failure was also the top-ranked predictor in both the permutation importance and SHAP analyses of Model 1.

History of myocardial infarction (36.6% vs. 23.5%, *p* = 0.010), a history of coronary artery disease/cardiac surgery (42.7% vs. 30.0%, *p* = 0.028), and preoperative cognitive impairment (36.6% vs. 23.7%, *p* = 0.018) were significantly more prevalent in the MINS group. Prior CABG was also more frequent in the MINS group (17.1% vs. 6.8%, *p* = 0.003), whereas documented prior PCI/stent (2.4% vs. 1.9%, *p* = 1.000), antiplatelet use (35.4% vs. 27.5%, *p* = 0.180) and anticoagulant use (18.3% vs. 13.8%, *p* = 0.354) did not differ significantly, statin use (17.1% vs. 10.3%, *p* = 0.101), also did not differ (Supplementary Table S1).

Preoperative hemoglobin [10.85 (IQR: 9.62–12.17) vs. 11.50 (IQR: 10.30–12.80) g/dL, *p* = 0.018] and albumin [33.50 (IQR: 30.30–36.52) vs. 35.20 (IQR: 31.10–38.20) g/L, *p* = 0.014] levels were significantly lower, while creatinine [1.02 (IQR: 0.79–1.33) vs. 0.87 (IQR: 0.69–1.11) mg/dL, *p* = 0.003] was significantly higher in the MINS group. These findings are summarized in Table 1.

Frailty burden was also significantly greater in the MINS group, as reflected in both mFI-5 (2.00 [1.00–3.00] vs. 2.00 [1.00–2.00], *p* = 0.010) and mFI-11 (3.50 [2.00–5.00] vs. 3.00 [1.00–4.00], *p* = 0.004) scores, as well as the RCRI score (1.00 [0.00–2.00] vs. 1.00 [0.00–1.00], *p* = 0.011).

Intraoperative vasopressor use (22.0% vs. 7.3%, *p* < 0.001) and postoperative mechanical ventilation requirement (9.8% vs. 2.6%, *p* = 0.004) were also significantly more common among patients with MINS. No significant differences were observed between the groups with respect to sex, ASA score, type of surgery, diabetes, or hypertension (*p* > 0.05 for all). MINS developed in 12.5% *(n* = 82) of the study population.

*Model 1 Performance and Comparative Analysis*

Only preoperative variables were included in this model. According to the VIF > 5.0 rule, 9 variables were excluded from the analysis, and following iterative importance-based selection of the remaining 27 candidates, 9 variables ultimately remained in the model. Among the models trained on the remaining preoperative variables after multicollinearity removal, the class-weighted Nearest Centroid algorithm exhibited the highest performance.

In the internal hold-out test set, Model 1 achieved an AUC of 0.6644 (95% CI: 0.5505–0.7805), a sensitivity of 0.5200, and a specificity of 0.7849. The optimal clinical decision threshold was determined as 0.119 using the Youden Index. The model showed a numerically higher AUC than the ASA score (AUC: 0.5267), with a difference of +0.1377 (DeLong Z = 2.0552, raw *p* = 0.040), although this difference did not retain statistical significance after Bonferroni correction across all 15 comparisons (adjusted *p* = 0.598); the difference of +0.0543 compared with RCRI (AUC: 0.6101) did not reach statistical significance (DeLong Z = 0.8909, *p* = 0.373; adjusted *p* = 1.000) (Figure 2).

In the decision curve analysis, Model 1 showed potential net benefit at thresholds of approximately 2–20% com-pared with the “treat all” and “treat none” strategies; at thresholds above 23%, the net benefit became negative (Figure 3).

*Model 2 Performance and Comparative Analysis*

Following the exclusion of mFI-5 components (hypertension, diabetes mellitus, congestive heart failure, COPD/pneumonia, functional dependency) and redundant, non-preoperative variables (mFI-11, VP/MV requirement, ASA, RCRI), 9 variables were removed from the analysis according to the VIF > 5.0 rule; after iterative variable selection, 10 variables remained in the final model, including the mFI-5 score. In this model, albumin (VIF = 13.54) was among the nine variables removed in the multicollinearity step (VIF > 5.0). The mFI-5 score, as the core study variable, was exempt from VIF-based elimination. Among the models trained with these variables, the class-weighted Nearest Centroid algorithm again exhibited the highest performance.

On the internal hold-out test set, Model 2 achieved an AUC of 0.6005 (95% CI: 0.4727–0.7236), with a sensitivity of 0.6400 and a specificity of 0.5756. The optimal clinical decision threshold was determined as 0.111 using the Youden Index. The model did not reach statistical significance relative to the ASA score (AUC: 0.5267), with a difference of +0.0738 (DeLong *p* = 0.261: adj *p* = 1.000); the difference relative to RCRI (AUC: 0.6101), at −0.0096, was likewise not significant (DeLong *p* = 0.885;adj *p* = 1.000) (Figure 2).

In decision curve analysis, Model 2 provided a limited positive net benefit compared with the “intervene for all” and “intervene for none” strategies only within a narrow, low threshold probability range (approximately a 11% threshold); no sustained net clinical benefit was observed within clinically meaningful threshold ranges (Figure 3).

*Model 3 Performance and Comparative Analysis*

In the third model, the mFI-11 frailty index was added to the preoperative variables; the components constituting the index were excluded from the model to prevent redundant variable use. Based on the VIF > 5.0 threshold, only 3 variables (age, hip surgery, preoperative hemoglobin) were eliminated, following these elimination steps, a total of 6 variables remained in the final model, and the CatBoostClassifier (unweighted) algorithm demonstrated the highest performance.

In the internal hold-out test set, Model 3 achieved an AUC of 0.6935 (95% CI: 0.5828–0.8052), with a sensitivity of 0.6400 and a specificity of 0.7500. The optimal clinical decision threshold was determined as 0.138 using the Youden Index. The model showed a numerically higher AUC than the ASA score (AUC: 0.5267), with a difference of +0.1667 (DeLong Z = 2.6385, raw *p* = 0.008), although this difference did not retain statistical significance after Bonferroni correction across all 15 comparisons (adjusted *p* = 0.125); the observed difference of +0.0834 relative to RCRI (AUC: 0.6101) did not reach statistical significance (DeLong Z = 1.5098, *p* = 0.131; adjusted *p* = 1.000) (Figure 2). The mFI-11 score, creatinine, and albumin emerged as the most determinant variables in the model’s decision mechanism, whereas the contributions of inhaler use, emergency surgery status, and digitalis use remained comparatively limited (Figure 4).

In the decision curve analysis, Model 3 showed potential net benefit within a limited threshold probability range of approximately 8–20% compared with the “treat all” and “treat none” strategies; above this range (approximately 22–30%), the net benefit turned negative (Figure 3).

To compare the discriminative performance of the frailty-based preoperative model configurations with the baseline preoperative model, paired DeLong tests were performed between Model 1 and Model 2 and between Model 1 and Model 3 on the same internal hold-out test set. The AUC difference for Model 2 relative to Model 1 was −0.0640 (Z = −1.504, *p* = 0.133; Bonferroni-adjusted *p* = 1.000), and the difference was not statistically significant. The AUC difference for Model 3 relative to Model 1 was found to be +0.0291 (Z = 0.578, *p* = 0.563; Bonferroni-corrected *p* = 1.000), and this difference likewise did not reach statistical significance (Table 2; full pairwise results with raw and Bonferroni-adjusted *p*-values are presented in Supplementary Table S2).

*Incremental Contribution of Frailty Indices: Sensitivity Analysis with Comparable Models*

To isolate the incremental contribution of frailty indices independent of variable selection and algorithm choice, three comparable models (baseline, +mFI-5, +mFI-11) were developed using the same classifier (class-weighted Nearest Centroid) and the same variable set (Supplementary Table S3; Supplementary Figures S1–S3). On the internal hold-out test set, the comparable baseline model achieved an AUC of 0.5744 (95% CI 0.4253–0.7132). It should be acknowledged that the comparable base model achieved only modest discrimination (AUC 0.574; 95% CI 0.425–0.713, which includes 0.5); the small incremental gains should therefore be interpreted cautiously. Adding mFI-5 yielded a numerically slightly higher AUC of 0.5828 (95% CI 0.4428–0.7233; ΔAUC = +0.0084), and adding mFI-11 yielded an AUC of 0.5881 (95% CI 0.4429–0.7240; ΔAUC = +0.0137). None of the pairwise differences reached statistical significance (baseline vs. +mFI-5: Z = −0.4493, *p* = 0.653; baseline vs. +mFI-11: Z = −0.6112, *p* = 0.541; +mFI-5 vs. +mFI-11: Z = −0.3607, *p* = 0.718). Brier scores were similar across all three models (0.1089–0.1098), and the differences between cross-validation and hold-out performance were small (0.0063–0.0141). Because the small event count limited the power to detect subtle differences, these findings neither establish nor exclude an incremental contribution of the frailty indices; the performance differences between the primary models may partly reflect differences in variable selection and algorithm choice, rather than the frailty indices alone.

*Model 4 Performance and Comparative Analysis*

In the fourth model, designed as an exploratory perioperative model, intraoperative (vasopressor requirement) and postoperative (mechanical ventilator requirement) variables were added alongside the preoperative variables; 10 variables were excluded from the analysis based on the VIF > 5.0 rule. Total of 12 variables remained in the final model. The CatBoostClassifier algorithm, which can accommodate class imbalance, achieved the highest performance, and the effect of each variable on the prediction was evaluated using SHAP analysis.

The model showed a numerically higher AUC over the ASA score (AUC: 0.5267), with a difference of +0.2316 (DeLong *p* < 0.001; Bonferroni-adjusted *p* = 0.002), and over RCRI (AUC: 0.6101), with a difference of +0.1483 (DeLong *p* = 0.018), although the latter difference did not retain statistical significance after Bonferroni correction (adjusted *p* = 0.268) (Figure 2).

In the decision curve analysis, Model 4 showed greater net benefit than the “treat-all” and “treat-none” strategies within the 8–30% threshold range; net benefit declined at higher thresholds. Because the model incorporates intraoperative and postoperative data, it does not offer preoperative decision support and should be regarded as an exploratory analysis (Figure 3).

In addition, calibration curves were evaluated for all four models (Figure 5). The calibration intercept and slope (95% CI) were −0.76 (−2.18 to 1.07) and 0.62 (−0.07 to 1.63) for Model 1; −0.80 (−2.97 to 1.14) and 0.57 (−0.49 to 1.59) for Model 2; 0.68 (−0.91 to 2.43) and 1.37 (0.55 to 2.34) for Model 3; and 0.36 (−0.93 to 1.69) and 1.24 (0.64 to 1.96) for Model 4, respectively. The positive calibration intercepts of Models 3 and 4 (0.68 and 0.36) indicate a tendency to underestimate risk, and the negative intercepts of Models 1 and 2 (−0.76 and −0.80) a tendency to overestimate risk; none of the four models achieved a calibration intercept close to zero. Calibration slopes deviated from unity in both directions (0.57–0.62 in Models 1–2; 1.24–1.37 in Models 3–4), and, given the small number of test-set events, all calibration metrics should be interpreted descriptively (Figure 5).

Model performances evaluated on the hold-out test set are summarized in Table 3. The exploratory perioperative model (Model 4) achieved the highest overall predictive accuracy, driven largely by intraoperative hemodynamic support requirements. After Bonferroni correction across all 15 pairwise comparisons (Supplementary Table S2), only the exploratory perioperative model (Model 4) numerically higher AUC than ASA physical status classification (adjusted *p* = 0.002); the advantages of Models 1 and 3 over ASA did not retain statistical significance (adjusted *p* = 0.598 and 0.125, respectively), and no model significantly outperformed the RCRI.

In addition to permutation importance, SHAP analysis was performed to evaluate the impact and direction of key variables on model predictions. The three top-ranked variables based on mean absolute SHAP values for each model are summarized in Table 4 and the full SHAP beeswarm distribution for the best-performing preoperative model (Model 3) is presented in (Figure 4).

## 4. Discussion

In this study, the discriminative performance of sequentially constructed preoperative machine learning models incorporating frailty indices was compared against a baseline model, in order to investigate the preoperative predictability of postoperative MINS in geriatric patients undergoing major orthopedic surgery. The baseline model (Model 1), which included only routine preoperative variables, demonstrated a numerically higher AUC than the ASA score—although this difference did not retain statistical significance after Bonferroni correction—and showed no comparable difference against the RCRI; in this cohort, the baseline preoperative model showed higher discrimination than ASA; however, this finding should be interpreted cautiously given the limited discrimination of ASA and the exploratory nature of these comparisons. The ASA showed limited discrimination in this cohort (AUC 0.5267), setting a very low bar. In contrast RCRI showed higher discrimination than ASA in this cohort (AUC 0.6101). Testing against the RCRI was therefore the far more relevant benchmark for incremental clinical benefit. Given that our model did not significantly outperform the RCRI (raw *p* = 0.131; adjusted *p* = 1.000), our findings should be interpreted with caution. While the addition of mFI-5 to the baseline model reduced its predictive performance, the model incorporating mFI-11 achieved the numerically highest discrimination among the preoperative models (AUC 0.69), although this improvement did not reach statistical significance relative to the baseline model and its advantage over ASA (raw *p* = 0.008) did not retain statistical significance after Bonferroni correction (adjusted *p* = 0.125). Our findings suggest that the contribution of frailty indices to the model may vary depending on the index used. However, whether a broader index provides a clinically meaningful advantage over baseline variables remains inconclusive. This uncertainty largely stems from the differing variable selection and algorithm choices between the models, as well as the limited sample size of our study. To address this uncertainty, we performed an additional sensitivity analysis in which the classifier and variable set were held constant and only the frailty index was varied (Supplementary Table S3). Within this directly comparable framework, adding mFI-5 and mFI-11 to the base model yielded only small, statistically non-significant increases in AUC (ΔAUC = +0.0084, *p* = 0.653 and ΔAUC = +0.0137, *p* = 0.541, respectively). These findings warrant cautious interpretation: given the modest discrimination of the baseline model and the statistical constraints of the sample size, the data neither definitively establish nor rule out an incremental contribution of the frailty indices.

MINS often follows a silent course. It has been reported in the literature to increase postoperative mortality by approximately five-fold [3] to be responsible for one-quarter of postoperative deaths [8], and to increase the risk of in-hospital mortality by 1.37–2.28-fold in hip fracture cases [10]. Given this silent course and its contribution to mortality, accurately identifying at-risk patients before surgery is of major clinical importance. Current guidelines strongly recommend MINS screening in high-risk patients; however, risk tools capable of performing this screening accurately, rapidly, and widely are still lacking [20]. Therefore, unlike classical risk scores, which focus solely on a patient’s existing comorbidities, it is important to determine whether frailty scales—which also reflect overall physiological reserve—contribute to MINS risk prediction.

mFI-11 measures perioperative frailty by combining 11 comorbidity and functional impairment components. Higher scores have been reported to be associated with adverse postoperative outcomes in various surgical populations [8,21]. This association appears to be more pronounced in orthopedic surgical groups [22,23]. mFI-11 has been reported to be directly associated with complications and mortality in patients undergoing hip arthroplasty [22], and in another cohort undergoing hip fracture surgery, increasing frailty scores have been shown to significantly increase the risk of one-year mortality [23]. In the meta-analysis by Dong et al., frailty was shown to be an independent and strong risk factor that increases both in-hospital and short- and long-term mortality by approximately twofold in this patient group [24].

There are numerous studies in the literature, using different frailty measurement tools, regarding the superiority of frailty-based scoring systems over traditional risk scores. Choi et al. [25] reported that a multidimensional frailty index was more successful in predicting 6-month mortality than ASA score and chronological age in geriatric patients operated for hip fracture, increasing the mortality risk by approximately 3.5-fold in the high-risk group. Although components such as cognitive status, mobility, nutrition, and polypharmacy do not fully align with the mFI-11 used in our study, our findings are compatible with this literature, even if they do not definitively confirm it. Further studies are warranted to determine the extent to which frailty assessment truly adds value beyond routine clinical tests for evaluating surgical risk. However, further studies are needed to determine just how much value it truly adds beyond routine clinical tests.

In our study, Model 3, based on mFI-11, achieved the highest absolute AUC value (0.6935) among the preoperative configurations examined and demonstrated a numerically higher AUC than the ASA classification (AUC: 0.5267; raw *p* = 0.008), although this difference did not retain statistical significance after Bonferroni correction (adjusted *p* = 0.125); the observed increase in AUC compared with the baseline model (Model 1) also did not reach statistical significance (Bonferroni-adjusted *p* = 1.000). In the SHAP analysis, the mFI-11 score showed the largest mean absolute SHAP contribution to Model 3 predictions, exceeding that of creatinine and albumin (Table 4). However, this did not yield a significant improvement in AUC compared with Model 1 or RCRI. This is likely due to an overlap with the cardiac parameters already present in the baseline model.

While the notable SHAP contribution indicates that mFI-11 strongly influenced Model 3’s predictions, this should not be taken as evidence that it offers superior independent prognostic value or provides a distinct incremental gain in discriminative power.

mFI-5 is a frailty scale widely used in surgical practice due to its ease of calculation from routine preoperative data [14]. It has been shown in the literature to predict mortality and overall complications in geriatric surgical populations [14]; it has also been reported to be successful in predicting in-hospital, 3-month, and 1-year mortality in geriatric patients with femoral fractures [26]. Although a significant association was found between mFI-5 and MINS in univariate analysis (*p* = 0.010), the addition of this variable to the model decreased the test AUC (from 0.6644 to 0.6005). This indicates that the negative permutation importance score of mFI-5 (−0.0035) reflects an increase in model noise rather than a contribution to predictive power in the test set. Because mFI-5 introduced multicollinearity (VIF > 5.0), we had to exclude albumin —an independently significant predictor (*p* = 0.014)—during variable selection. This drop in performance might stem more from differences in the resulting predictor sets across models than from a direct causal impact of removing albumin. For this reason, mFI-5 may have caused a loss of information by displacing other strong predictors. Unlike mFI-5, the addition of mFI-11 did not lead to the elimination of albumin specifically, despite VIF-based removal of three other variables (age, hip surgery, preoperative hemoglobin) from Model 3. This finding is also consistent with the comparable-model sensitivity analysis. When the variable set was held constant, the model including mFI-5 did not perform worse than the comparable base model (AUC 0.5828 vs. 0.5744; DeLong *p* = 0.653). Accordingly, the lower performance of Model 2 in the primary analysis may be related, at least in part, to the exclusion of potentially informative variables such as albumin; however, as the differences in the sensitivity analysis were not statistically significant, this explanation remains speculative and a direct contribution of the frailty score cannot be excluded. This finding indicates that frailty scores valid for mortality and morbidity in surgical cases cannot be directly extrapolated to specific organ injuries such as MINS, and that simple comorbidity-based indices may be insufficient on their own for perioperative cardiac risk stratification.

Few studies have directly compared mFI-5 and mFI-11. In direct comparisons, both indices showed comparable outcome prediction [27,28]. For example, in elderly spine surgery patients, the c-statistics of both indices were similar for major complications (0.68 vs. 0.68) and 6-month mortality (0.83 vs. 0.81) [28]. In a trauma registry, both indices were likewise associated with complications and discharge disposition, although the correlation between them was lower in older patients and in the isolated hip fracture subgroup than in other cohorts, and neither index predicted mortality [27]. A meta-analysis in elective spine surgery noted that mFI-5 showed more consistent associations across various clinical outcomes, though the authors emphasized that this comparison remained indirect rather than head-to-head, and that mFI-11 could be pooled for only three outcomes [29]. In our study, mFI-11 yielded numerically higher discrimination for predicting MINS than mFI-5 (AUC 0.6935 vs. 0.6005; ΔAUC = +0.093, 95% CI −0.009 to 0.195; DeLong Z = 1.781, raw *p* = 0.075; Bonferroni-adjusted *p* = 1.000); however, this difference did not reach statistical significance, and both models showed limited discrimination (AUC < 0.70). The comparison is also subject to confounding because the models were built using different variable sets and algorithms. This finding may suggest that frailty indices evaluated for general postoperative outcomes do not perform equivalently for a specific endpoint such as MINS. One possible explanation is that mFI-11 includes additional cardiovascular items (myocardial infarction, prior PMI/cardiac surgery, peripheral vascular disease, TIA/stroke) that are absent from mFI-5 and may be more relevant to a cardiac endpoint. This remains a hypothesis, underscoring the need for dedicated, direct comparative study.

RCRI is one of the risk tools developed for perioperative cardiac events and frequently used in MINS prediction [30]. ASA physical status classification, on the other hand, is a non-cardiac-specific assessment of the patient’s suitability for anesthesia and does not represent the pathophysiological processes underlying MINS (ischemic heart disease, congestive heart failure, renal dysfunction). A study by Bronheim et al. showed that ASA’s performance in predicting major cardiac events remains markedly lower compared with RCRI [31]. In our study, the models we developed showed numerically higher AUCs than ASA, although only the raw *p*-values reached nominal significance before correction (Model 1: *p* = 0.040 vs. ASA and *p* = 0.373 vs. RCRI; Model 3: *p* = 0.008 vs. ASA and *p* = 0.131 vs. RCRI). These comparator analyses were exploratory and were corrected for multiplicity across all 15 pairwise comparisons (Supplementary Table S2); the Model 1 vs. ASA result (raw *p* = 0.040) did not retain statistical significance after correction (adjusted *p* = 0.598) and in particular should not be interpreted as a robust finding. Structural differences between the two scores play an important role in this discrepancy. Nearly all of the components comprising RCRI are also core components of our preoperative models. In contrast, since no such structural overlap exists with ASA, the difference emerges more clearly. Because CHF, creatinine, and mFI-11—the parameters that stand out in the SHAP analyses of Model 1 and Model 3, respectively—overlap substantially with RCRI, the discriminative contribution that the preoperative models add over RCRI has remained limited. This structural overlap aligns with the finding that Model 4—which incorporates perioperative hemodynamic stress (vasopressor use and mechanical ventilation) and thus shares fewer components with RCRI—achieved numerically higher AUC over ASA (Bonferroni-adjusted *p* = 0.002) but not over RCRI (Bonferroni-adjusted *p* = 0.268). However, since our study included only 82 MINS events, it should be noted that all DeLong comparisons—including those showing significant differences against ASA—were conducted with limited power, resulting in wide confidence intervals for effect size estimates. Therefore, rather than attributing the RCRI result solely to insufficient statistical power, both RCRI and ASA comparisons should be interpreted with this limitation in mind.

The similarity between our model and the RCRI likely reflects the fact that both rely on shared cardiac variables, rather than a genuine lack of added value. Even so, this remains a plausible explanation rather than a proven fact, and it should definitely not be interpreted as evidence that Model 3 outperforms the RCRI.

Current perioperative guidelines recommend integrating clinical risk scores with functional capacity and cardiac biomarkers; the incremental value of biomarkers over the RCRI has been well documented in the literature [6]. However, due to the retrospective design of our study, NT-proBNP/BNP levels and objective functional capacity assessments were not available; consequently, the models could only be compared against the RCRI and the ASA physical status classification. This is a relevant limitation, as conventional cardiac risk scores have shown limited discrimination for postoperative cardiovascular events in external populations, including a recent prospective Brazilian cohort in which the RCRI and ACP scores yielded AUCs below 0.70 [32].

Model 4, incorporating intraoperative vasopressor use and postoperative mechanical ventilation requirement, provided the highest discriminative ability in our study (AUC 0.7584, sensitivity 0.8000, specificity 0.6686); It was the only model with a Bonferroni-adjusted *p* below 0.05 against ASA after Bonferroni correction (adjusted *p* = 0.002); its difference against RCRI did not retain statistical significance after correction (adjusted *p* = 0.268). However, this model cannot be used directly as a preoperative decision-making model. Vasopressor use and postoperative mechanical ventilation requirement, which are included in the model, are variables that cannot be known before surgery. Intraoperative vasopressor requirement is a direct indicator of acute hemodynamic deterioration and the oxygen supply–demand imbalance underlying MINS [30]. The fact that the model’s net clinical benefit in decision curve analysis remains confined to a narrow threshold range (8–30%) further supports this limitation. Therefore, Model 4 should be interpreted solely as an exploratory analysis illustrating the additional predictive information contained in perioperative physiological deterioration, rather than as a preoperative risk-stratification model [33].

According to the decision curve analysis, the preoperative models provided positive net benefit only within narrow, model-dependent threshold ranges: Model 1 at approximately 2–20% (turning negative beyond 23%), Model 3 at approximately 8–20% (negative between 22 and 30%), and Model 2 only at a single threshold (~11%), without a sustained range of benefit. Because these estimates derive from a hold-out set of only ~25 events and are not accompanied by confidence intervals, they are imprecise and should not be taken to indicate clinical utility at any threshold. These findings are hypothesis-generating only, and no clinical use of these models is supported until they have been externally and prospectively validated.

Nevertheless, the decline of net benefit into negative values at higher thresholds indicates that preoperative models alone are not sufficient to identify high-risk patients, and these models should not be used as decision-support tools at this stage, on other hand, the fact that Model 4, which incorporates intraoperative and postoperative variables (vasopressor and mechanical ventilation requirement), provided benefit across a wider range (8–30%) demonstrates that discriminative power increases only when perioperative processes are also taken into account. However, since these variables cannot be known preoperatively, Model 4 cannot be used as a preoperative screening tool; rather, it should be regarded solely as a finding that underscores the importance of the risk imposed by the surgical process and the intraoperative course.

The cross-validation mean AUCs (0.57–0.67) indicate modest discrimination on average across folds. In Models 1, 3 and 4, the hold-out AUC was higher than the CV mean, whereas Model 2 showed a small opposite gap (+0.0241). Given that the hold-out set contained only ~25 MINS events, both estimates are imprecise and consistent with limited, rather than strong, discrimination. The higher hold-out values are therefore more probably an artifact of sampling variability in the small test cohort than evidence of better generalization; since the hold-out set was not used at any stage of model development, information leakage is unlikely.

## 5. Limitations

The single-center, retrospective design carries a risk of selection bias, and the absence of independent external vali-dation limits the generalizability of the findings to different populations. Only patients admitted to the intensive care unit (ICU) after surgery and monitored with routine clinical care were included in the study. Because both criteria reflect clinical decisions made for high-risk patients, our findings may not be generalizable to geriatric orthopedic patients who are managed without such surveillance. The reported MINS incidence (12.5%) should be interpreted within this context and cannot be directly extrapolated to the broader geriatric orthopedic population. Of the 800 screened patients, 144 (18%) were excluded (53 with baseline hs-cTnT ≥ 65 ng/L, 19 with dialysis-dependent end-stage renal disease, and 72 with missing critical data); the excluded patients may differ systematically from the analyzed cohort, and the direction and magnitude of any resulting selection bias are unknown.

In a retrospective, single-center design, it is difficult to reliably distinguish acute perioperative injury from chronic myocardial injury in patients with elevated baseline values, which can compromise the reliability of outcome ascertainment. We therefore excluded patients with a baseline hs-cTnT ≥ 65 ng/L (*n* = 53). Although MINS defined by a dynamic troponin rise can also develop in these patients, fixed VISION thresholds cannot reliably detect acute in-jury against a chronically elevated baseline. Our models should therefore be applied only to patients with a baseline hs-cTnT below 65 ng/L and without dialysis-dependent end-stage renal disease, and their performance outside this subgroup is unknown.

Because of the retrospective design, the timing of perioperative interruption of antiplatelet therapy, stent type, and time since PCI could not be assessed; therefore, we could not evaluate whether antiplatelet discontinuation contributed to MINS. Prior PCI/stent was documented in only 13 patients and may be under-ascertained. Preoperative NT-proBNP/BNP levels and objective functional capacity assessments (such as DASI or METs) were unavailable, precluding a direct comparison with guideline-recommended pathways. Future prospective studies should compare machine learning models against the guideline-recommended approach, which combines a clinical risk score, an objective assessment of functional capacity (DASI or METs), and preoperative NT-proBNP/BNP measurement, to determine whether the models add prognostic value beyond this integrated approach.

Because no prior power calculation was performed, as all eligible consecutive ICU patients during the study period were included, the number of MINS cases (*n* = 82) represents the natural yield of the study period rather than a pre-determined sample size. Although conventional events-per-variable criteria were originally established for regression models and do not directly translate to all machine learning approaches, the limited number of MINS events remains an important constraint on model complexity, potentially compromising the stability of more complex algorithms. The number of models and parameter combinations evaluated was also relatively large compared with the number of outcome events. Although the internal hold-out test set remained completely untouched during model development, the preliminary algorithm-screening step preceded the nested cross-validation procedure and may have introduced some residual model-selection optimism. The study was also not powered to detect differences between the models and the comparator scores; the wide confidence intervals around the AUC estimates (Table 3) apply equally to significant and non-significant comparisons, so the results should not be over-interpreted. The comparative performance of the models should be interpreted cautiously and confirmed in larger external cohorts.

Calibration metrics were computed on the internal hold-out test set, which contained only approximately 25 MINS events; therefore, the calibration intercept and slope estimates have wide confidence intervals and should be interpreted cautiously. The deviation of the calibration slopes from unity (0.57–0.62 in Models 1–2 and 1.24–1.37 in Models 3–4) is probably attributable, at least in part, to this small sample size. Models 1, 3, and 4 produced very similar optimal decision thresholds, falling in a narrow 12–14% risk range (0.119, 0.138, and 0.117, respectively). However, because these cut-offs rely on Youden’s index from a single-center cohort with only about 25 test-set events, the sample is too small to recommend them for routine screening. They would first need validation in larger, multicenter cohorts.

Because variable selection and algorithm choice were re-optimized for each model, the AUC differences between Model 1 and Models 2–3 reflect the combined effect of the frailty index, the updated variable set, and the chosen algorithm—not the isolated impact of the frailty index itself. While this approach mirrors practical clinical workflow development, it prevents isolating the exact performance gain attributable to frailty indices. The sensitivity analysis with a fixed classifier and a fixed variable set showed only a small incremental contribution, which could not be reliably estimated because of low power. It was limited to Nearest Centroid, leaving the incremental value of mFI-11 within a gradient-boosting framework unassessed; this finding remains hypothesis-generating. Finally, Bonferroni adjustment was applied across all 15 pairwise comparisons among the six candidates (Models 1–4, the ASA physical status classification, and RCRI); because this conservative correction reduces statistical power, the reported significance levels should be interpreted cautiously.

Independent external validation studies encompassing different centers, populations, and surgical fields are needed for these findings to be translated into clinical practice; the current results obtained from the internal test set are, at this stage, only hypothesis-generating. Future studies with larger samples, external and prospective validation, improved calibration, and inclusion of guideline-recommended components (objective functional capacity, NT-proBNP/BNP) are required to determine whether mFI-11-based models have any incremental value; until such validation is available, no clinical use of these models is recommended.

## 6. Conclusions

This study evaluated the contribution of modified frailty indices to predicting preoperative MINS risk in geriatric orthopedic patients. Among the preoperative models, the mFI-11–based model (Model 3) had the highest hold-out AUC (0.6935), but it did not show statistically significant superiority over standard risk scores (RCRI and ASA) after multiple testing correction. Because Models 2 and 3 differed in both predictors and algorithm, this difference cannot be attributed to the frailty index alone, and in the sensitivity analysis with an identical classifier and predictor set no demonstrable incremental value was observed for either index. Consequently, an advantage of mFI-based approaches over existing validated clinical scores could not be established, and our findings remain exploratory. External validation in larger, independent cohorts is required before these models can be translated into routine clinical practice.

## Figures and Tables

**Figure 1:**
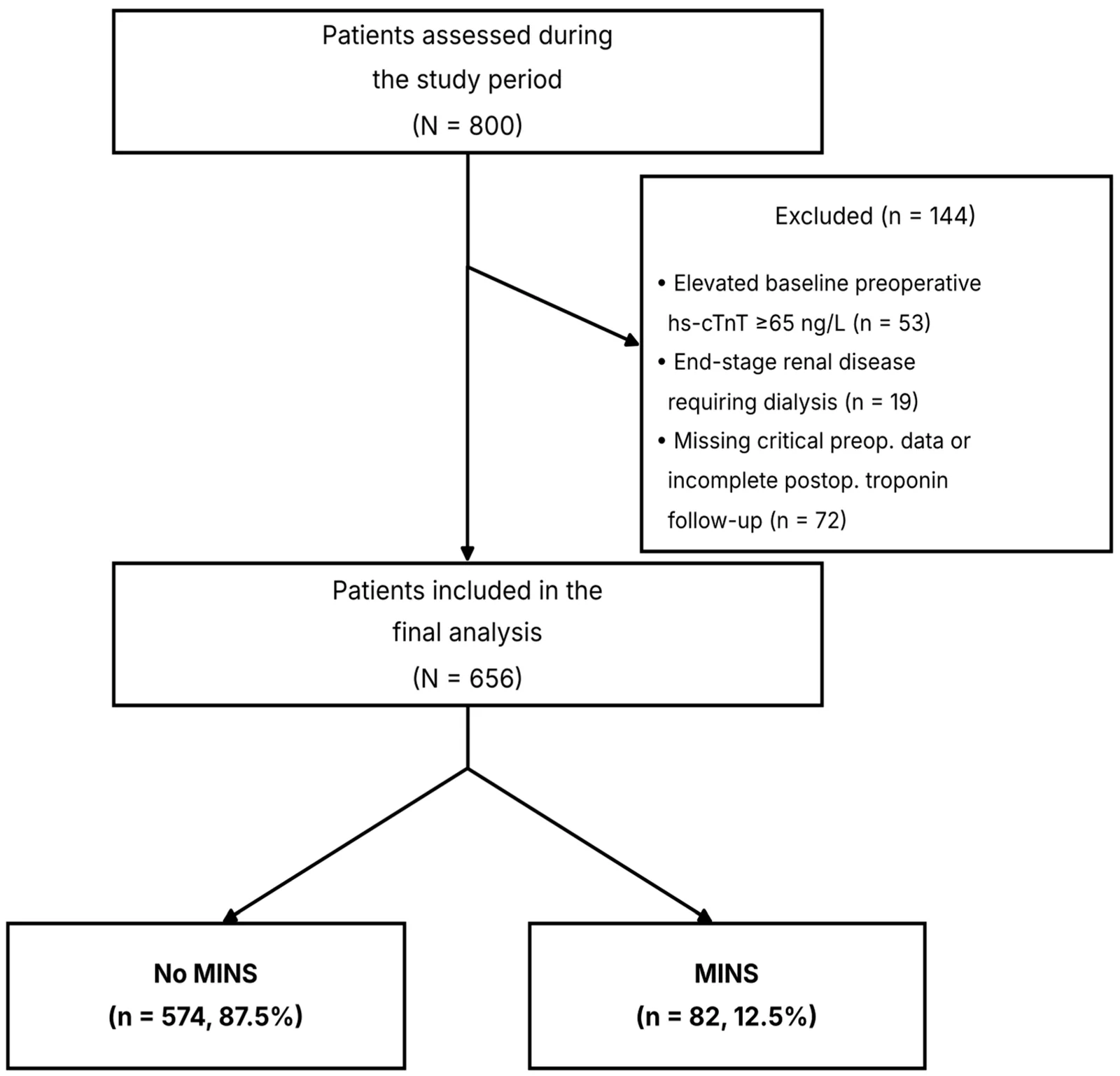
Patient flow diagram.

**Figure 2:**
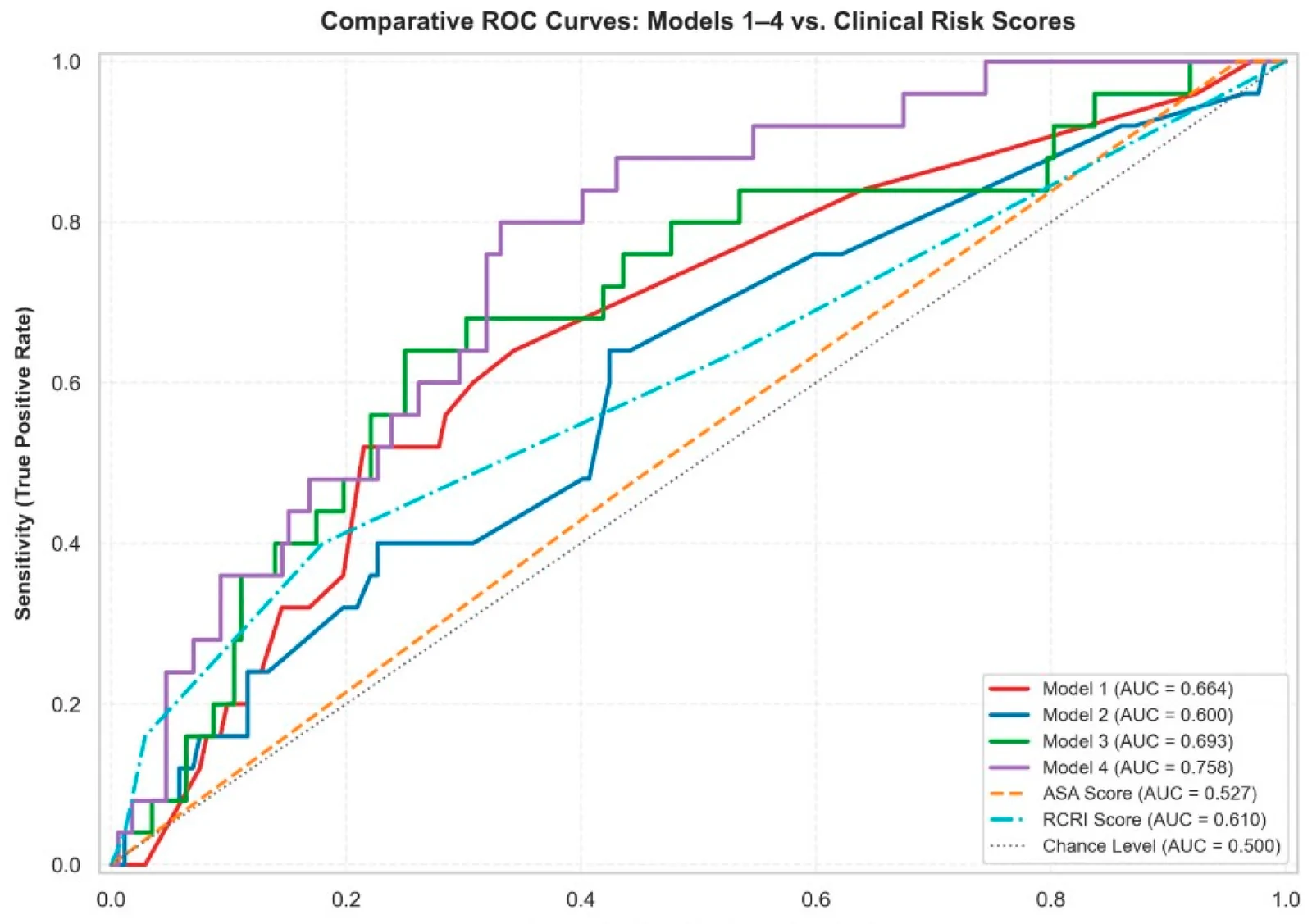
Comparative receiver operating characteristic (ROC) curves for the four machine learning models (Model 1–4) alongside the standalone ASA physical status classification and the Revised Cardiac Risk Index (RCRI) for predicting myocardial injury after non-cardiac surgery (MINS), evaluated on the internal hold-out test set. Model 4 (AUC = 0.758), the exploratory model incorporating intraoperative and postoperative variables, achieved the highest discriminative performance, followed by Model 3 (AUC = 0.693), Model 1 (AUC = 0.664), and Model 2 (AUC = 0.600). Both ASA (AUC = 0.527) and RCRI (AUC = 0.610) are shown for reference, together with the diagonal line of no discrimination (chance level, AUC = 0.500.

**Figure 3:**
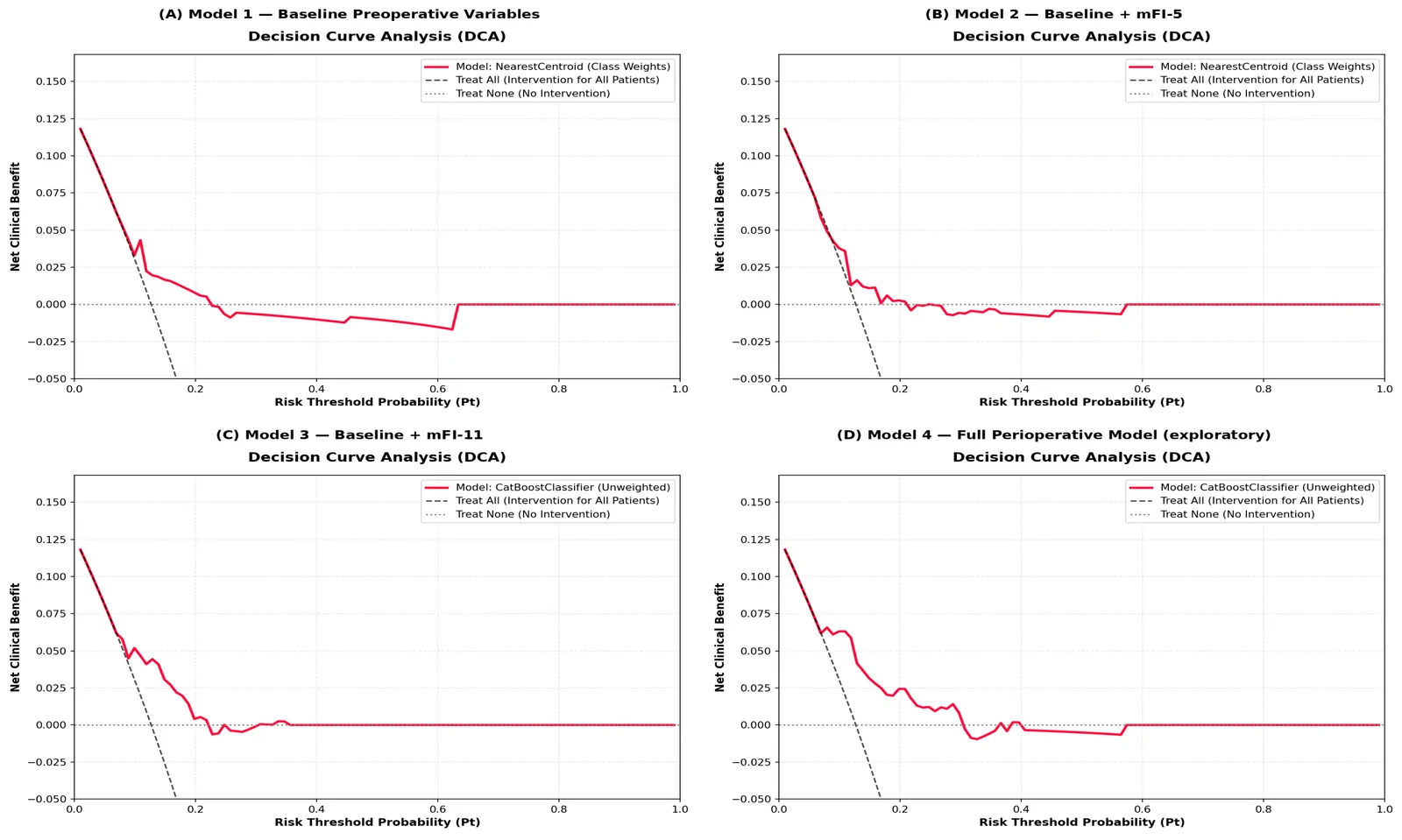
Decision Curve Analysis for Models 1–4. (**A**) Model 1 (baseline preoperative variables, NearestCentroid classifier) shows potential net clinical benefit at low decision thresholds, with the net benefit approaching zero at approximately 0.20–0.22 and becoming negative at higher thresholds. (**B**) Model 2 (baseline + mFI-5) shows a similarly modest benefit that flattens near zero shortly after the 0.10–0.20 threshold range, offering little advantage over Treat None across most of the threshold spectrum. (**C**) Model 3 (mFI-11-based preoperative model, CatBoostClassifier) shows potential net benefit within an approximate threshold range of 8–20%, followed by negative net benefit at approximately 22–30% before converging with Treat None, consistent with its performance among the preoperative-only models. (**D**) Model 4 (full perioperative model including intraoperative/postoperative variables, exploratory) shows the highest net benefit at low-to-moderate thresholds (up to ~0.30) but with a similar early convergence pattern, consistent with its exploratory rather than confirmatory role. Across all panels, the red curve represents the respective model, the dashed line represents the “Treat All” strategy, and the dotted line represents the “Treat None” strategy.

**Figure 4:**
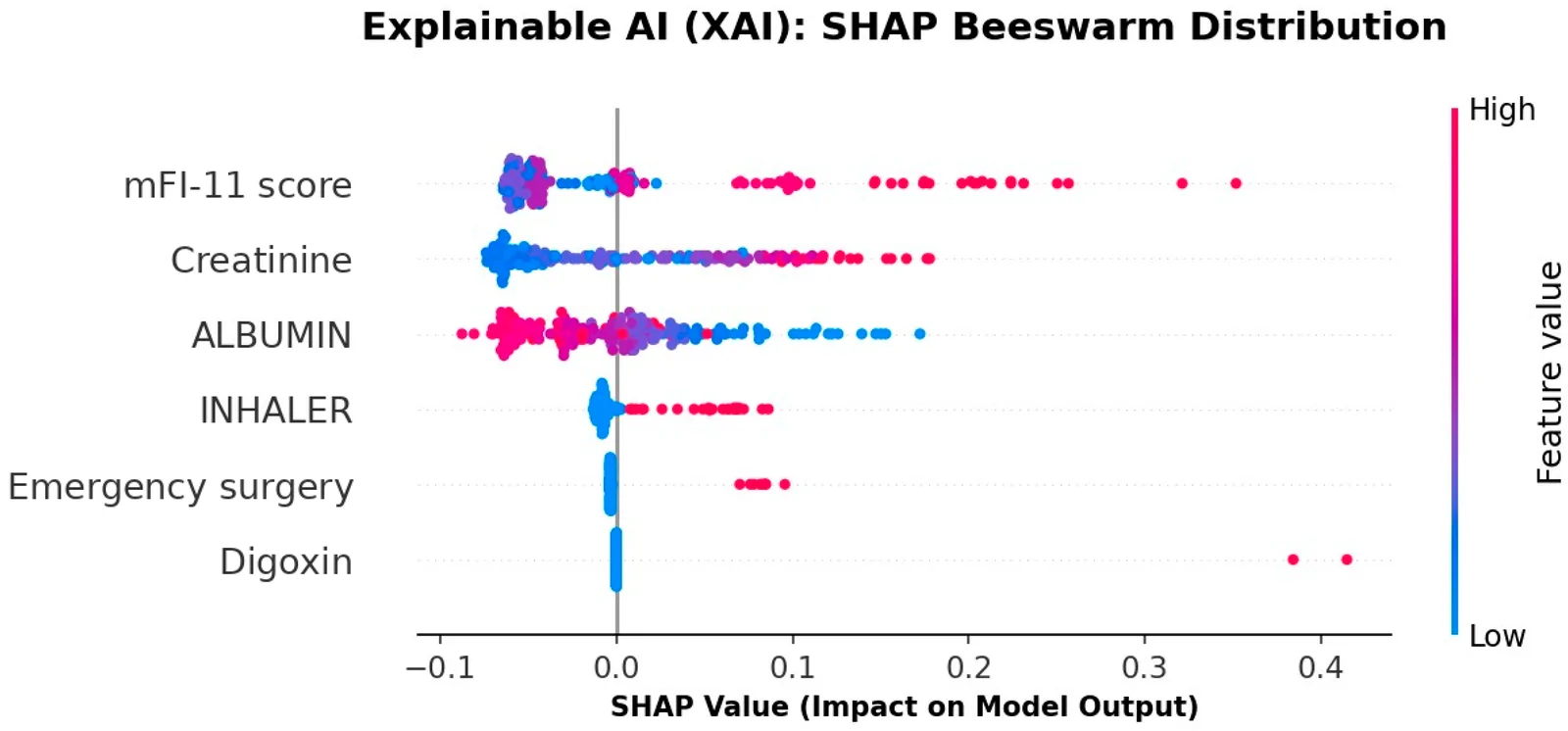
SHAP beeswarm plot for Model 3 (mFI-11–based CatBoostClassifier), computed on the 30% internal hold-out test set. Each point represents an individual patient; the horizontal position indicates the SHAP value (impact on model output toward higher predicted MINS risk), and color denotes the corresponding feature value (blue = low, red/pink = high). Variables are ordered from top to bottom by mean absolute SHAP value. The mFI-11 score showed the greatest overall contribution to model output, followed by creatinine and albumin; inhaler use, emergency surgery status, and Digitalis use contributed comparatively less to the model’s predictions, although digitalis use produced two high-impact outlier predictions.

**Figure 5:**
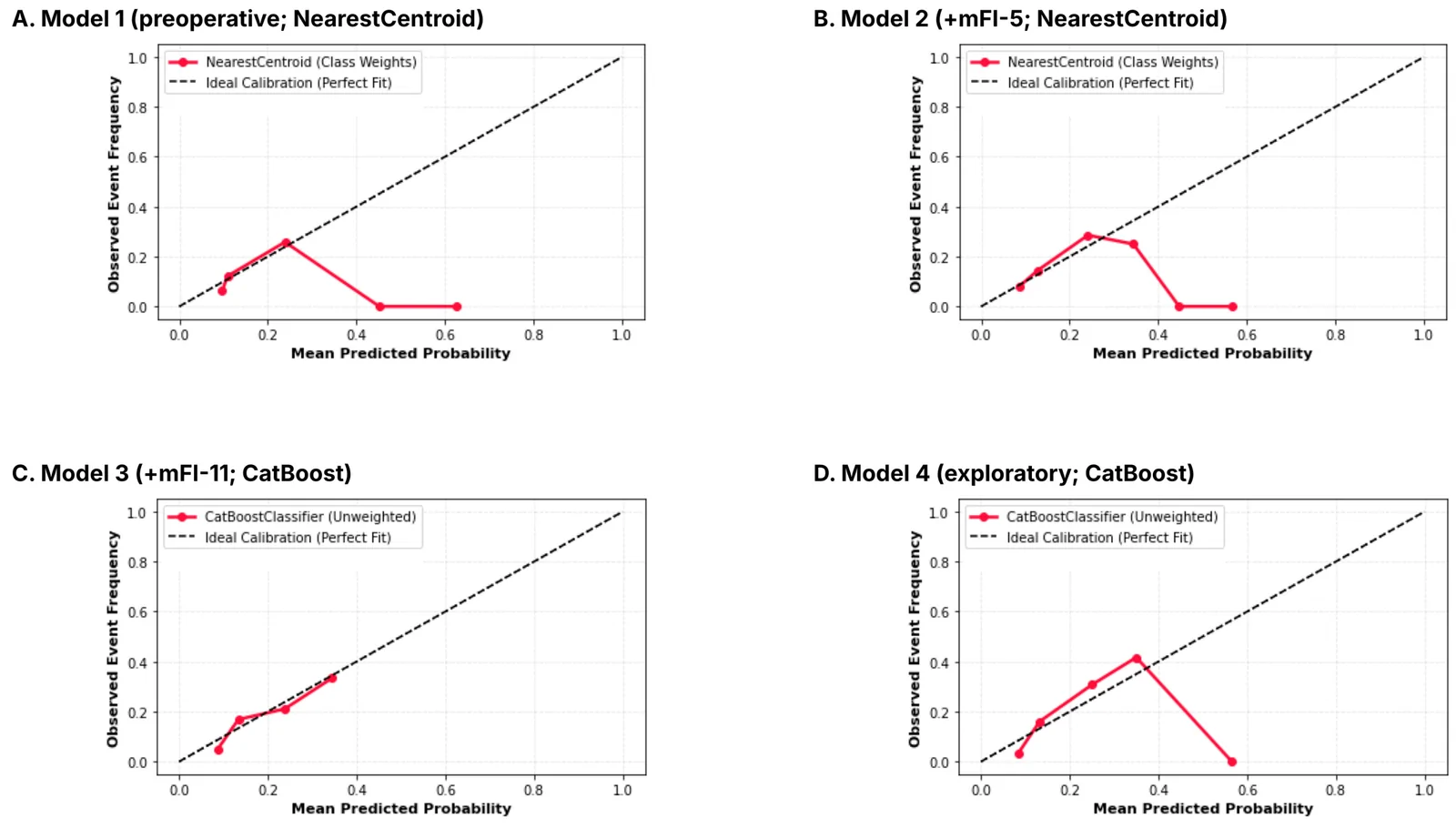
Calibration curves for the four primary models evaluated on the internal hold-out test set: (**A**) Model 1 (baseline preoperative model; class-weighted Nearest Centroid), (**B**) Model 2 (baseline + mFI-5; class-weighted Nearest Centroid), (**C**) Model 3 (baseline + mFI-11; CatBoostClassifier, unweighted), (**D**) Model 4 (exploratory perioperative model including vasopressor and mechanical ventilation requirements; CatBoostClassifier, unweighted). The diagonal dashed line represents perfect calibration. Calibration intercept and slope (95% CI) for each model are reported in the Section 3: (**A**) −0.76 (−2.18 to 1.07) and 0.62 (−0.07 to 1.63); (**B**) −0.80 (−2.97 to 1.14) and 0.57 (−0.49 to 1.59); (**C**) 0.68 (−0.91 to 2.43) and 1.37 (0.55 to 2.34); (**D**) 0.36 (−0.93 to 1.69) and 1.24 (0.64 to 1.96).

**Table 1:** Baseline demographic and clinical characteristics of patients with and without myocardial injury after noncardiac surgery (MINS).

Variable	MINS (−) (*n* = 574)	MINS (+) (*n* = 82)	*p*
Age (years)	78.00 (72.00–83.00)	80.50 (73.00–86.00)	0.048
Female sex, n (%)	376 (65.5)	54 (65.9)	0.981
ASA score	3.00 (3.00–4.00)	4.00 (3.00–4.00)	0.060
Emergency surgery, n (%)	28 (4.9)	4 (4.9)	1.000
Hip surgery, n (%)	465 (81.0)	72 (87.8)	0.180
Upper extremity surgery, n (%)	58 (10.1)	9 (11.0)	0.816
Knee surgery, n (%)	35 (6.1)	2 (2.4)	0.197
Lower extremity surgery, n (%)	21 (3.7)	2 (2.4)	0.762
Malignancy surgery, n (%)	60 (10.5)	7 (8.5)	0.634
Diabetes mellitus, n (%)	198 (34.5)	32 (39.0)	0.453
Hypertension, n (%)	375 (65.3)	59 (72.0)	0.246
Congestive heart failure, n (%)	65 (11.3)	23 (28.0)	<0.001
Prior myocardial infarction, n (%)	135 (23.5)	30 (36.6)	0.010
Cardiac surgery/Coronary artery disease, n (%)	172 (30.0)	35 (42.7)	0.028
Prior CABG, n (%)	39 (6.8)	14 (17.1)	0.003
Documented prior PCI/stent, n (%)	11 (1.9)	2 (2.4)	1.000
Antiplatelet use, n (%)	158 (27.5)	29 (35.4)	0.180
Clopidogrel use, n (%)	32 (5.6)	7 (8.5)	0.417
Anticoagulant use, n (%)	79 (13.8)	15 (18.3)	0.354
Peripheral vascular disease, n (%)	10 (1.7)	4 (4.9)	0.085
Cognitive impairment, n (%)	136 (23.7)	30 (36.6)	0.018
Cerebrovascular disease, n (%)	46 (8.0)	4 (4.9)	0.349
Prior stroke, n (%)	42 (7.3)	3 (3.7)	0.238
COPD/Pneumonia, n (%)	88 (15.3)	10 (12.2)	0.487
Functional dependency, n (%)	289 (50.3)	50 (61.0)	0.092
mFI-5 score	2.00 (1.00–2.00)	2.00 (1.00–3.00)	0.010
mFI-11 score	3.00 (1.00–4.00)	3.50 (2.00–5.00)	0.004
Preoperative hemoglobin (g/dL)	11.50 (10.30–12.80)	10.85 (9.62–12.17)	0.018
Creatinine (mg/dL)	0.87 (0.69–1.11)	1.02 (0.79–1.33)	0.003
Albumin (g/L)	35.20 (31.10–38.20)	33.50 (30.30–36.52)	0.014
RCRI score	1.00 (0.00–1.00)	1.00 (0.00–2.00)	0.011

Data are presented as median (interquartile range) for continuous variables and as n (%) for categorical variables. Continuous variables were compared using the Mann–Whitney U test; categorical variables were compared using the chi-square test or Fisher’s exact test, as appropriate. ASA, American Society of Anesthesiologists physical status classification; COPD, chronic obstructive pulmonary disease; MINS, myocardial injury after noncardiac surgery; mFI-5, 5-item modified frailty index; mFI-11, 11-item modified frailty index; RCRI, Revised Cardiac Risk Index. CABG, coronary artery bypass grafting; PCI, percutaneous coronary intervention. Prior CABG and PCI/stent were identified from free-text comorbidity records; PCI/stent was counted only when explicitly documented and may be under-ascertained. Medication data reflect preoperative records; the timing of perioperative interruption was not recorded.

**Table 2:** Pairwise DeLong Comparisons of Model Discrimination (AUC).

Model A	Model B	AUC (A)	AUC (B)	AUC Difference (A-B)	Z Score	*p* Value	Bonferroni-Corrected *p*	Significant After Bonferroni Correction (Adjusted *p*
Model 1	Model 2	0.6644	0.6005	+0.0640	1.504	0.1326	1.0000	No
Model 1	Model 3	0.6644	0.6935	−0.0291	−0.578	0.5632	1.000	No
Model 1	Model 4	0.6644	0.7584	−0.0940	−1.576	0.1151	1.0000	No
Model 2	Model 3	0.6005	0.6935	−0.0930	−1.781	0.0750	1.0000	No
Model 2	Model 4	0.6005	0.7584	−0.1579	−2.349	0.0188	0.282	No
Model 3	Model 4	0.6935	0.7584	−0.0649	−1.262	0.2068	1.000	No

Bonferroni-corrected *p* values were adjusted across all 15 pairwise comparisons among the six candidates (Models 1–4, the ASA physical status classification, and RCRI) and capped at a maximum of 1.000; the full set of 15 pairwise comparisons with raw and adjusted *p*-values is presented in Supplementary Table S2.

**Table 3:** Performance Summary of Machine Learning Models and Top Variables by Permutation Importance.

Model/Algorithm	Most Important Variables (Perm. Importance)	CV Mean AUC	Holdout AUC (95% CI)	Brier	Sens./Spec.	ΔAUC vs. ASA/RCRI (*p*)
Model 1 (Baseline Preoperative) NearestCentroid	CHF (0.0647) Statin use (0.0527) Knee surgery (0.0234)	0.5682	0.6644 (0.5505–0.7805)	0.1121	Sens: 0.5200 Spec: 0.7849	ASA: +0.1377 (*p* = 0.040; adj. *p* = 0.598) RCRI: +0.0543 (*p* = 0.373; adj. *p* = 1.000)
Model 2 (+mFI-5) NearestCentroid	Cognitive impairment (0.0570) Peripheral vascular disease (0.0459) Insulin use (0.0068)	0.6246	0.6005 (0.4727–0.7236)	0.1114	Sens: 0.6400 Spec: 0.5756	ASA: +0.0738 (*p* = 0.261; adj. *p* = 1.000) RCRI: −0.0096 (*p* = 0.885; adj. *p* = 1.000)
Model 3 (+mFI-11) CatBoostClassifier	Creatinine (0.0637) Digitalis use (0.0534) Inhaler (0.0364) mFI-11 score (0.0303)	0.5677	0.6935 (0.5828–0.8052)	0.1061	Sens: 0.6400 Spec: 0.7500	ASA: +0.1667 (*p* = 0.008; adj. *p* = 0.125) RCRI: +0.0834 (*p* = 0.131; adj. *p* = 1.000)
Model 4 (+VP/MV, Exploratory) CatBoostClassifier	Insulin use (0.0414) Vasopressor requirement (0.0390) Antiparkinsonian medication use (0.0363)	0.6727	0.7584 (0.6661–0.8407)	0.1036	Sens: 0.8000 Spec: 0.6686	ASA: +0.2316 (*p* ≤ 0.001; adj. *p* = 0.002) RCRI: +0.1483 (*p* = 0.018; adj. *p* = 0.268)

Abbreviations: AUC, area under the receiver operating characteristic curve; CV, cross-validation; CI, confidence interval; ASA, American Society of Anesthesiologists physical status classification; RCRI, Revised Cardiac Risk Index; CHF, congestive heart failure; mFI-5, 5-item modified frailty index; mFI-11, 11-item modified frailty index; VP, vasopressor; MV, mechanical ventilation. CV Mean AUC denotes the mean ROC-AUC across the outer folds of the 5-fold nested cross-validation performed on the training subset; Holdout AUC denotes performance on the internal hold-out test set.

**Table 4:** Top Three Predictors by Mean Absolute SHAP Value for Each Model.

Model	Rank 1	Rank 2	Rank 3
Model 1 (Baseline Preoperative)	CHF (0.0316)	Peripheral vascular disease (0.0051)	Anticoagulant use (0.0050)
Model 2 (+mFI-5)	Cognitive impairment (0.0145)	Peripheral vascular disease (0.0141)	Insulin use (0.0076)
Model 3 (+mFI-11)	mFI-11 score (0.0622)	Creatinine (0.0574)	Albumin (0.0403)
Model 4 (+VP/MV, Exploratory)	Vasopressor requirement (0.0539)	Creatinine (0.0406)	Albumin (0.0353)

Values represent mean absolute SHAP (SHapley Additive exPlanations) values computed on the 30% hold-out test set for each model, reflecting each variable’s average contribution magnitude to individual model predictions. mFI, modified frailty index; VP, vasopressor; MV, mechanical ventilation.

## Data Availability

The data presented in this study are available on reasonable request from the corresponding author. The data are not publicly available due to privacy and ethical restrictions.

## Supplementary Materials

The following supporting information can be downloaded at: https://www.mdpi.com/article/10.3390/jcm15218285/s1, Figure S1: Baseline model (class-weighted Nearest Centroid, 25 preoperative variables, no frailty index) from the sensitivity analysis in which the classifier and variable set were held constant: (A) ROC curve on the internal hold-out test set, (B) decision curve analysis, (C) native feature weights; Figure S2: Baseline + mFI-5 model (class-weighted Nearest Centroid, 26 variables) from the sensitivity analysis: (A) ROC curve, (B) decision curve analysis, (C) native feature weights; Figure S3: Baseline + mFI-11 model (class-weighted Nearest Centroid, 26 variables) from the sensitivity analysis: (A) ROC curve, (B) decision curve analysis, (C) native feature weights; Table S1: Preoperative medication use in patients with and without myocardial injury after noncardiac surgery (MINS); Table S2: Pairwise DeLong comparisons of discrimination (AUC) among Models 1–4, the ASA physical status classification and RCRI on the internal hold-out test set; Table S3: Incremental contribution of frailty indices: comparable models using the same classifier and the same candidate predictor pool.

## Abbreviations

ASA	American Society of Anesthesiologists
AUC	Area Under the Curve
BMI	Body Mass Index
CI	Confidence Interval
DCA	Decision Curve Analysis
ECG	Electrocardiography
ICU	Intensive Care Unit
IQR	Interquartile Range
mFI-5	5-item Modified Frailty Index
mFI-11	11-item Modified Frailty Index
MINS	Myocardial Injury After Non-Cardiac Surgery
ML	Machine Learning
NPV	Negative Predictive Value
PCA	Principal Component Analysis
PPV	Positive Predictive Value
RCRI	Revised Cardiac Risk Index
ROC	Receiver Operating Characteristic
SHAP	SHapley Additive exPlanations
VIF	Variance Inflation Factor
