# Supplementary material for: Frailty Indices and Preoperative Clinical Factors for Predicting Myocardial Injury After Non-Cardiac Surgery (MINS) in High-Risk Geriatric Orthopedic Patients: A Machine-Learning Study
Source: J Clin Med. 2026 Oct 24;15(21):8285. doi: 10.3390/jcm15218285 (PMC13764412; doi:10.3390/jcm15218285)
Supplement: Supplementary file 1 [file jcm-15-08285-s001.zip › Supplementary_Table_S1.pdf]

Table S1. Preoperative medication use in patients with and without myocardial injury after noncardiac surgery (MINS)

Variable	MINS (–) (n = 574)	MINS (+) (n = 82)	P
Antihypertensive	403 (70.2)	65 (79.3)	0.117
Diuretic	168 (29.3)	27 (32.9)	0.583
Digitalis	5 (0.9)	3 (3.7)	0.066
Antianginal	34 (5.9)	6 (7.3)	0.805
Statin	59 (10.3)	14 (17.1)	0.101
Antiplatelet	158 (27.5)	29 (35.4)	0.180
Anticoagulant	79 (13.8)	15 (18.3)	0.354
Insulin	55 (9.6)	13 (15.9)	0.121
Oral antidiabetic	137 (23.9)	18 (22.0)	0.808
Inhaler	88 (15.3)	10 (12.2)	0.562
Antidepressant	32 (5.6)	3 (3.7)	0.606
Antipsychotic	36 (6.3)	4 (4.9)	0.805
Antiparkinsonian	39 (6.8)	3 (3.7)	0.399
Antiepileptic	10 (1.7)	0 (0.0)	0.622

Data are presented as n (%). Groups were compared using the chi-square test or Fisher's exact test, as appropriate. MINS, myocardial injury after noncardiac surgery. Medication data reflect preoperative records; doses, the timing of perioperative interruption and postoperative MINS treatment were not recorded. Antihypertensive agents were recorded as a single group; beta-blockers and ACE inhibitors/ARBs were not recorded separately.
