# Supplementary material for: Frailty Indices and Preoperative Clinical Factors for Predicting Myocardial Injury After Non-Cardiac Surgery (MINS) in High-Risk Geriatric Orthopedic Patients: A Machine-Learning Study
Source: J Clin Med. 2026 Oct 24;15(21):8285. doi: 10.3390/jcm15218285 (PMC13764412; doi:10.3390/jcm15218285)
Supplement: Supplementary file 1 [file jcm-15-08285-s001.zip › Supplementary_Table_S2.pdf]

Table S2. Pairwise DeLong comparisons of discrimination (AUC) among Models 1–4, the ASA physical status classification and RCRI on the internal hold-out test set

Candidate A	Candidate B	AUC (A)	AUC (B)	AUC Difference (A–B)	Z Score	Raw p	Bonferroni-adjusted p	Significant (adjusted p < 0.05)
Model 1	Model 2	0.6644	0.6005	+0.0640	1.504	0.1326	1.0000	No
Model 1	Model 3	0.6644	0.6935	−0.0291	−0.578	0.5632	1.0000	No
Model 1	Model 4	0.6644	0.7584	−0.0940	−1.576	0.1151	1.0000	No
Model 1	ASA	0.6644	0.5267	+0.1377	2.055	0.0399	0.5979	No
Model 1	RCRI	0.6644	0.6101	+0.0543	0.891	0.3730	1.0000	No
Model 2	Model 3	0.6005	0.6935	−0.0930	−1.781	0.0750	1.0000	No
Model 2	Model 4	0.6005	0.7584	−0.1579	−2.349	0.0188	0.2823	No
Model 2	ASA	0.6005	0.5267	+0.0737	1.125	0.2607	1.0000	No
Model 2	RCRI	0.6005	0.6101	−0.0097	−0.144	0.8854	1.0000	No
Model 3	Model 4	0.6935	0.7584	−0.0649	−1.262	0.2068	1.0000	No
Model 3	ASA	0.6935	0.5267	+0.1667	2.639	0.0083	0.1249	No
Model 3	RCRI	0.6935	0.6101	+0.0834	1.510	0.1311	1.0000	No
**Model 4**	**ASA**	**0.7584**	**0.5267**	**+0.2316**	**3.819**	**<0.001**	**0.0020**	**Yes**
Model 4	RCRI	0.7584	0.6101	+0.1483	2.369	0.0179	0.2679	No
ASA	RCRI	0.5267	0.6101	−0.0834	−1.126	0.2603	1.0000	No

Comparisons were performed with the paired DeLong test on the same internal hold-out test set. Bonferroni correction was applied across all 15 pairwise comparisons among the six candidates (Models 1–4, ASA and RCRI); adjusted p-values are capped at 1.000. AUC, area under the receiver operating characteristic curve; ASA, American Society of Anesthesiologists physical status classification; RCRI, Revised Cardiac Risk Index.
