# Supplementary material for: Frailty Indices and Preoperative Clinical Factors for Predicting Myocardial Injury After Non-Cardiac Surgery (MINS) in High-Risk Geriatric Orthopedic Patients: A Machine-Learning Study
Source: J Clin Med. 2026 Oct 24;15(21):8285. doi: 10.3390/jcm15218285 (PMC13764412; doi:10.3390/jcm15218285)
Supplement: Supplementary file 1 [file jcm-15-08285-s001.zip › Supplementary_Table_S3.pdf]

Table S3. Incremental contribution of frailty indices: comparable models using the same classifier and the same candidate predictor pool

Panel A. Performance on the internal hold-out test set

Model	No. of features	Test AUC (95% CI)	Sensitivity	Specificity	Brier score	Overfit gap
Comparable base model	25	0.5744 (0.4253–0.7132)	0.480	0.767	0.1098	0.0141
Base model + mFI-5	26	0.5828 (0.4428–0.7233)	0.440	0.756	0.1096	0.0063
Base model + mFI-11	26	0.5881 (0.4429–0.7240)	0.400	0.814	0.1089	0.0082

Panel B. Pairwise paired DeLong comparisons

Model A	Model B	AUC (A)	AUC (B)	AUC difference (A–B)	Z score	p value
Comparable base model	Base + mFI-5	0.5744	0.5828	−0.0084	−0.449	0.653
Comparable base model	Base + mFI-11	0.5744	0.5881	−0.0137	−0.611	0.541
Base + mFI-5	Base + mFI-11	0.5828	0.5881	−0.0053	−0.361	0.718

All three models used the same classifier (class-weighted Nearest Centroid) and the same candidate predictor pool; the only difference between the models was the inclusion of no frailty index (comparable base model), the mFI-5 score (+mFI-5) or the mFI-11 score (+mFI-11). To keep the predictor set identical, VIF-based exclusion and importance-based iterative feature selection were intentionally omitted; consequently, these models contain 25–26 variables and are diagnostic models designed to isolate the contribution of the frailty index, not candidate clinical models. Nested cross-validation, preprocessing and probability calibration were identical to those of the primary models. All values were obtained on the same internal hold-out test set (approximately 25 MINS events). AUC, area under the receiver operating characteristic curve; CI, confidence interval (bootstrap, 1000 iterations); mFI, modified frailty index. Overfit gap, absolute difference between cross-validation and hold-out performance. Differences between AUC values were assessed with the paired DeLong test; none was statistically significant. Given the small number of events, the CIs are wide and the analysis had limited power to detect subtle differences.
